# Histamine H_1_ receptor on astrocytes and neurons controls distinct aspects of mouse behaviour

**DOI:** 10.1038/s41598-019-52623-6

**Published:** 2019-11-11

**Authors:** Anikó Kárpáti, Takeo Yoshikawa, Fumito Naganuma, Takuro Matsuzawa, Haruna Kitano, Yo Yamada, Mariko Yokoyama, Akira Futatsugi, Katsuhiko Mikoshiba, Kazuhiko Yanai

**Affiliations:** 1 Department of Pharmacology, Tohoku University Graduate School of Medicine, 2-1 Seiryo-machi, Aoba-ku, Sendai, 980-8575 Japan; 2 Division of Pharmacology, Faculty of Medicine, Tohoku Medical and Pharmaceutical University, 1-15-1 Fukumuro, Miyagino-ku, Sendai, 983-8536 Japan; 3 Department of Basic Medical Sciences, Kobe City College of Nursing, 3-4 Gakuen-nishi-machi, Nishi-ku, Kobe, 651-2103 Japan; 4 Shanghai Institute for Advanced Immunochemical Studies, ShanghaiTech University, Shanghai, 201210 China

**Keywords:** Cognitive neuroscience, Astrocyte

## Abstract

Histamine is an important neurotransmitter that contributes to various processes, including the sleep-wake cycle, learning, memory, and stress responses. Its actions are mediated through histamine H_1_–H_4_ receptors. Gene knockout and pharmacological studies have revealed the importance of H_1_ receptors in learning and memory, regulation of aggression, and wakefulness. H_1_ receptors are abundantly expressed on neurons and astrocytes. However, to date, studies selectively investigating the roles of neuronal and astrocytic H_1_ receptors in behaviour are lacking. We generated novel astrocyte- and neuron-specific conditional knockout (cKO) mice to address this gap in knowledge. cKO mice showed cell-specific reduction of H_1_ receptor gene expression. Behavioural assessment revealed significant changes and highlighted the importance of H_1_ receptors on both astrocytes and neurons. H_1_ receptors on both cell types played a significant role in anxiety. Astrocytic H_1_ receptors were involved in regulating aggressive behaviour, circadian rhythms, and quality of wakefulness, but not sleep behaviour. Our results emphasise the roles of neuronal H_1_ receptors in recognition memory. In conclusion, this study highlights the novel roles of H_1_ receptors on astrocytes and neurons in various brain functions.

## Introduction

Histamine is an important monoamine neurotransmitter in the central nervous system (CNS). Histamine is released from histaminergic neurons residing in the tuberomammillary nucleus (TMN) that project throughout the brain. Histamine is involved in many essential physiological processes such as the sleep-wake cycle, anxiety, learning, memory, and stress responses^1–3^. Alterations in brain histamine levels are closely related to CNS dysfunction and are thought to contribute to various neurological disorders, including Alzheimer’s disease and depression^4^. Histamine binds to four distinct G protein-coupled receptors (GPCRs), termed histamine H_1_–H_4_ receptors. Previous studies have reported their functional expression in the CNS^5,6^. Conventional H_1_ receptor gene (*Hrh1*) knockout mice showed significant phenotypical differences compared to controls. For example, exploratory behaviour, aggression, learning, and memory were affected by *Hrh1* deletion^7–10^. Further, pharmacological studies have emphasised the importance of H_1_ receptors for wakefulness and cognition in humans^11,12^.

Although previous studies have provided crucial insights into the role of H_1_ receptor signalling in CNS functions, it remains unclear how H_1_ receptor-dependent signalling of different cell types contributes to a given phenotype. *Hrh1* is abundantly expressed in neurons^5^ and astrocytes^13,14^; however, until a few decades ago, the functions of astrocytes were poorly understood. Thus, research on the potential contribution of astrocytes to higher brain function was fairly neglected, and findings were traditionally attributed to neuronal signalling. Since then astrocytes have been comprehensively researched and are now accepted as multifunctional cells that critically contribute to CNS physiology^15^. With their end feet closely located at synapses, astrocytes are involved in cerebral blood flow^16^, energy metabolism^17^, ionic homoeostasis^18^, and synaptic function^19,20^. Growing evidence from rodent studies has stressed the importance of astrocyte signalling in complex behaviours, such as memory and circadian rhythm^21–23^. Furthermore, various neurological diseases, including Alzheimer’s disease and epilepsy, are associated with pathological states of astrocytes in rodents and humans^24,25^. Recent studies reported a role of H_1_ receptor-mediated astrocyte signalling in the regulation of glutamate release and clearance by astrocytes, release of inflammatory mediators from astrocytes, and astrocyte migration *in vitro*^14,26–28^. Collectively, compelling evidence demonstrates the multifarious functions of astrocytic H_1_ receptors *in vitro*, emphasising the need to selectively assess the impact of neuronal and astrocytic H_1_ receptor signalling on behaviour.

In this study, we developed novel conditional knockout (cKO) mice to selectively reduce *Hrh1* expression in either astrocytes or neurons. The aim of this study was to assess phenotypes of cKO mice and compare them to controls to better understand the impact of cell-specific H_1_ receptor signalling on brain function.

## Results

### Generation of astrocyte- and neuron-specific *Hrh1* cKO mice

**Figure 1:**
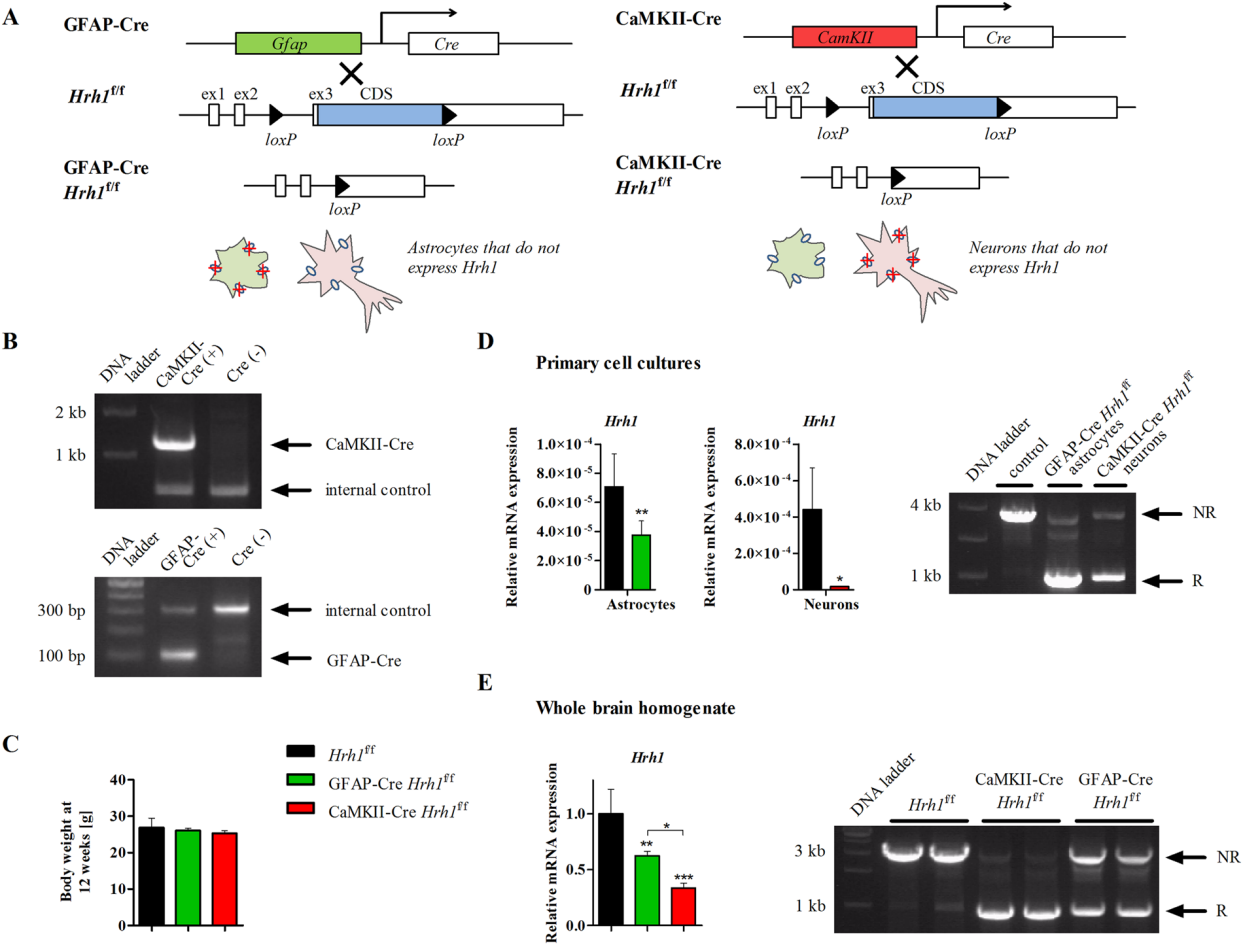
Generation and validation of conditional *Hrh1* knockout mice. (**A**) Schematic overview of the generation of novel conditional knockout (cKO) mice: GFAP-Cre *Hrh1*^f/f^ (left) and CaMKII-Cre *Hrh1*^f/f^ (right), were generated by crossing Cre recombinase-expressing mice with mice that carried *loxP* recognition sites at the *Hrh1* coding sequence (CDS) of exon 3 (ex3). (**B**) Representative PCR genotyping results of CaMKII-Cre (upper) or GFAP-Cre (lower) transgenic mice (cropped agarose gels are shown, see Supplementary Fig. S1 for full length gels). (**C**) Body weight was assessed at 12 weeks of age (n = 10–12). Data were analysed with one-way ANOVA and Tukey’s post-hoc test. (**D**) *Left: Hrh1* expression in primary astrocyte or neuron cell cultures was assessed with real-time PCR. Glyceraldehyde-3-phosphate dehydrogenase (*Gapdh*) served as an internal control (n = 3–6). Data were analysed with *t*-test (*p < 0.05 and **p < 0.01). *Right:* Visualisation of successful recombination at the *Hrh1 loxP* sites (cropped agarose gel is shown, see Supplementary Fig. S1 for full length gel). NR: no recombination (3244 bp), R: recombination (1106 bp). (**E**) *Left: Hrh1* expression in whole brain homogenates was assessed by real-time PCR. *Gapdh* served as an internal control (n = 6–7). Data were analysed with one-way ANOVA and Tukey’s post-hoc test (*p < 0.05, **p < 0.01 and ***p < 0.001). *Right:* Visualisation of successful recombination at the *Hrh1 loxP* sites (cropped agarose gel is shown, see Supplementary Fig. S1 for full length gel).

Novel astrocyte-specific glial fibrillary acidic protein- (GFAP) Cre *Hrh1*^f/f^ and neuron-specific Ca^2+^/calmodulin-dependent protein kinase II- (CaMKII) Cre *Hrh1*^f/f^ mice were successfully generated by crossing GFAP-Cre-^29^ or CaMKII-Cre-transgene^30^ positive mice with *Hrh1*^f/f^ mice that carried conditional *Hrh1* alleles; two *loxP* sites flanked the coding sequence of *Hrh1* in exon 3 (Fig. 1A). We confirmed the presence of the Cre transgene in genomic DNA of newly generated cKO mice by PCR (Fig. 1B and see Supplementary Fig. S1). Conditional *Hrh1* deletion did not lead to any obvious physical abnormalities; mice appeared healthy and of the same size as the control group. The fertility of the mice was not affected. Body weight of male mice was similar across the three study groups at 12 weeks (Fig. 1C). Conditional GFAP-Cre- and CaMKII-Cre-mediated gene deletion was indicated by successful excision of the targeted sequence and resulted in significantly reduced *Hrh1* expression levels, both in primary cell cultures and adult brains (Fig. 1D,E and see Supplementary Fig. S1). *Hrh1* mRNA expression was reduced by 47% in GFAP-Cre *Hrh1*^f/f^ astrocytes and by 96% in CaMKII-Cre *Hrh1*^f/f^ neurons compared to their respective controls obtained from *Hrh1*^f/f^ mice. Immunohistochemical analyses confirmed cell-specific Cre-recombinase expression. The results showed the presence of Cre-recombinase in GFAP-Cre *Hrh1*^f/f^ astrocytes and CaMKII-Cre *Hrh1*^f/f^ neurons, respectively (see Supplementary Fig. S2). We also confirmed *Hrh1* expression levels were not changed in other tissues of cKO mice (see Supplementary Fig. S3).

Concentrations of histamine, its metabolite 1-methylhistamine, and other monoamine neurotransmitters as well as their metabolites were measured in homogenates from various brain regions including the hypothalamus, hippocampus, prefrontal cortex, cortex, cerebellum, and diencephalon. Histamine concentrations remained unchanged in all brain regions except for the hypothalamus (Table 1). Here, the level of histamine was twice as high in CaMKII-Cre *Hrh1*^f/f^ mice than in controls. There were no changes in 1-methylhistamine concentrations in any brain region. Concentrations of all measured monoamines were consistent across all groups; however, the concentration of the dopamine metabolite 3,4-dihydroxyphenylacetic acid (DOPAC) was significantly increased in the prefrontal cortex (2-fold) and cerebellum (2.3-fold) of CaMKII-Cre *Hrh1*^f/f^ mice (see Supplementary Table S1). The ratio of dopamine turnover The turnover rate of dopamine, which was the sum of all dopamine metabolites, DOPAC, 3-MT, and HVA, divided by dopamine, was not affected by changes in DOPAC concentration.

**Table 1:** Concentrations of histamine and 1-methylhistamine in selected brain regions.

Brain region		Concentration [pM/mg tissue]		
		*Hrh1* ^f/f^	GFAP-Cre *Hrh1*^f/f^	CaMKII-Cre *Hrh1*^f/f^
Hypothalamus	HA	0.64 ± 0.301	0.84 ± 0.216	1.26 ± 0.502*
	1-MH	1.22 ± 0.409	0.99 ± 0.423	1.32 ± 0.412
Hippocampus	HA	0.09 ± 0.045	0.15 ± 0.044	0.22 ± 0.161
	1-MH	0.51 ± 0.104	0.52 ± 0.207	0.37 ± 0.097
Prefrontal cortex	HA	0.09 ± 0.024	0.28 ± 0.129	0.25 ± 0.212
	1-MH	0.48 ± 0.399	0.49 ± 0.369	0.56 ± 0.269
Cortex	HA	0.09 ± 0.012	0.13 ± 0.047	0.19 ± 0.15
	1-MH	0.23 ± 0.117	0.22 ± 0.131	0.19 ± 0.11
Cerebellum	HA	0.06 ± 0.032	0.09 ± 0.034	0.13 ± 0.117
	1-MH	0.08 ± 0.038	0.07 ± 0.076	0.16 ± 0.228
Diencephalon	HA	0.2 ± 0.047	0.26 ± 0.081	0.33 ± 0.119
	1-MH	0.62 ± 0.3	0.67 ± 0.211	0.71 ± 0.363

n = 6. Statistical differences among the groups were determined using one-way ANOVA with Tukey’s post-hoc test (*p < 0.05). HA: histamine, 1-MH: 1-methylhistamine.

### Neuronal H_1_ receptors are important for recognition memory

**Figure 2:**
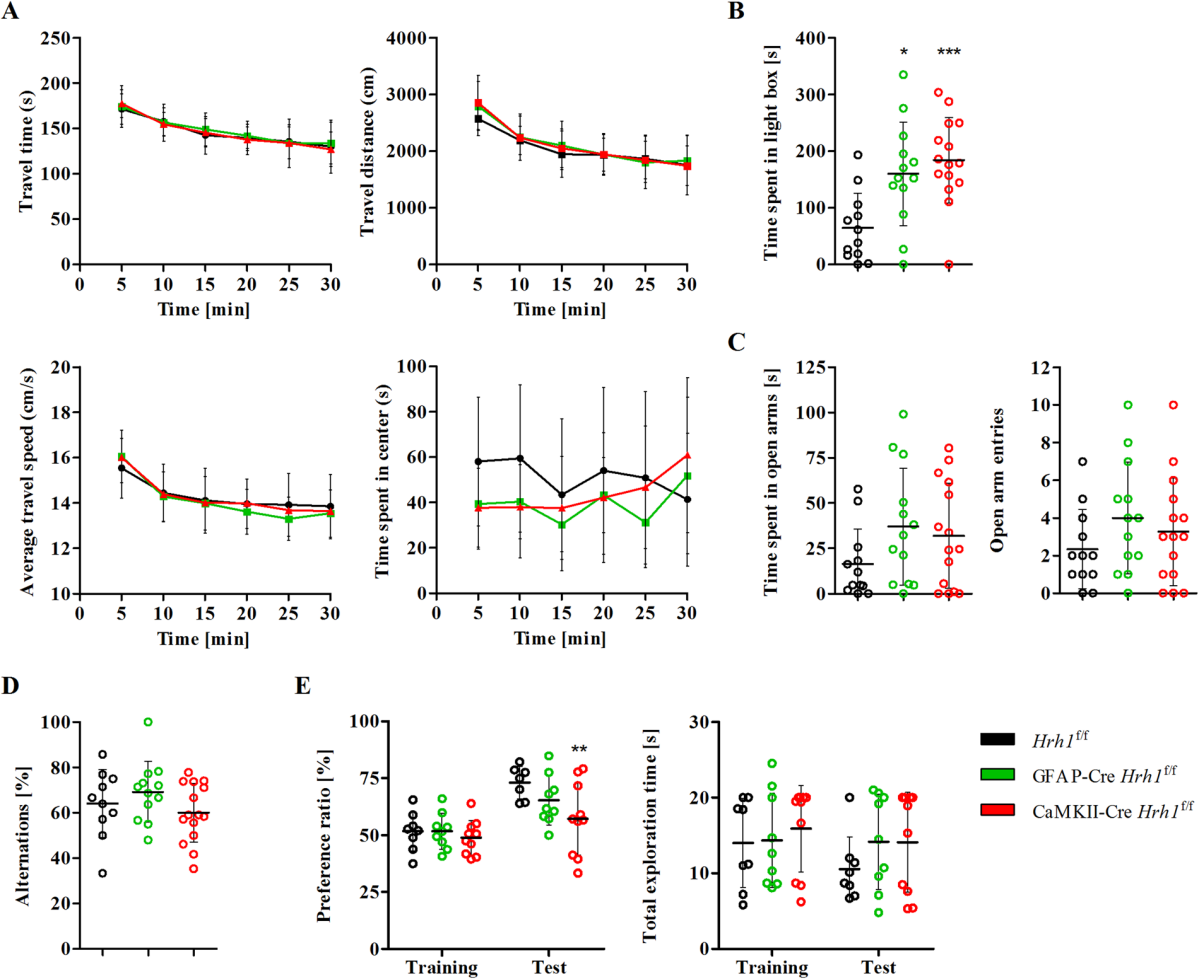
Behavioural assessment of cKO mice. (**A**) Exploratory behaviour and locomotor activity were assessed using the open-field test. Assessment of travel time, travel distance, average travel speed, and time spent in the centre of mice that moved freely in the open field for 30 min (n = 13–15). Two-way ANOVA and Bonferroni post-test were used for statistical analysis. (**B**) Light-dark box test was used to assess anxiety-like behaviour. After a 10 min session, the time spent in the light compartment was calculated (n = 12–15). Data were analysed with one-way ANOVA and Tukey’s post-hoc test (*p < 0.05 and ***p < 0.001). (**C**) Elevated plus-maze test was performed to assess anxiety-like behaviour. Mice were allowed to move freely on a cross-shaped platform for 10 min. The time spent in the open arms and number of arm entries were analysed (n = 12–15). Statistical differences were assessed with one-way ANOVA and Tukey’s post-hoc test. (**D**) Spatial memory was tested with the Y-maze test. Alternations were calculated after an 8-min session (n = 10–15). Data were analysed with one-way ANOVA and Tukey’s post-hoc test. (**E**) Recognition memory was assessed using the novel object recognition test. Mice were allowed to freely explore the environment and objects for 5 min. Cut-off time for total exploration was 20 s. Mice exhibiting decreased exploratory behaviour (<5 s of total exploration) were excluded from the statistical analysis (n = 5–10). Data were analysed with two-way ANOVA and Bonferroni post-hoc test (**p < 0.01).

It is established that histamine H_1_ receptor plays a role in exploratory behaviour, anxiety, and learning, and memory amongst others^7–10^. A behavioural test battery was set up to investigate potential phenotypic changes in cKO mice. First, cKO mice were subjected to the open-field test to test their exploratory behaviour, but no changes were observed (Fig. 2A). The travel time, travel distance, average travel speed, and time spent in the centre of the arena were similar among all groups. Anxiety-like behaviour was assessed in two additional tests: the light-dark box test (Fig. 2B) and elevated plus-maze test (Fig. 2C). During the light-dark box test, both GFAP-Cre *Hrh1*^f/f^ and CaMKII-Cre *Hrh1*^f/f^ mice spent significantly more time in the illuminated compartment, a measure of reduced anxiety. In the elevated plus-maze test, we did not observe a statistically significant change in anxiety-like behaviour between the groups.

To evaluate memory function in cKO mice, the spontaneous alternation (Y-maze) test and novel object recognition test were performed. The performance of cKO mice in the Y-maze test indicated that spatial learning was not affected by *Hrh1* deletion (Fig. 2D). In contrast, the outcome of the novel object recognition task suggested that recognition memory in mice lacking neuronal H_1_ receptors was impaired (Fig. 2E). This result is indicated by a significantly reduced preference ratio for CaMKII-Cre *Hrh1*^f/f^ mice during the test session compared to that for controls. The deletion of astrocytic H_1_ receptors had no impact on recognition memory. In summary, our results showed that exploratory behaviour was not affected by the conditional H_1_ receptor deletion. In contrast, anxiety-like behaviour was strongly decreased in both conditional KO mouse models and recognition memory was impaired in mice lacking the neuronal H_1_ receptor.

### Deletion of astrocytic H_1_ receptors increases aggressive behaviour

**Figure 3:**
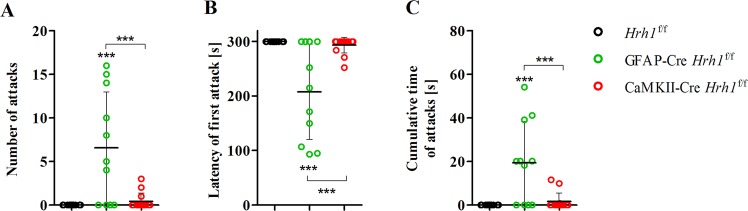
Astrocyte-specific knockout mice showed increased aggressive behaviour. Aggressive behaviour of male cKO and control mice was assessed using the resident-intruder test. Resident mice were confronted in their home cage with an unfamiliar mouse for 5 min. The number of attacks (**A**), latency until the first attack (**B**), and cumulative time of attacks (**C**) were analysed (n = 11–14). In case of no attack, the latency of first attack was set to 300 s and cumulative time of attacks was set to 0. Statistical significance was assessed with one-way ANOVA and Tukey’s post-hoc test (***p < 0.001).

Previously, a link between H_1_ receptors and aggression in conventional *Hrh1* KO mice was shown^8^. We studied cKO mice to attribute the regulation of aggressive behaviour to H_1_ receptors on either astrocytes or neurons, respectively. Unfamiliar intruders were never attacked by resident *Hrh1*^f/f^ controls, but GFAP-Cre *Hrh1*^f/f^ residents showed aggressive behaviour in the presence of an unfamiliar intruder (Fig. 3). Within an encounter of 5 minutes, GFAP-Cre *Hrh1*^f/f^ mice attacked intruders up to 15 times (Fig. 3A). On average, the latency of the first attack was 3.5 min, with some mice attacking the intruder for the first time within the first 2 minutes (Fig. 3B). The average cumulative attack time was 20 s for GFAP-Cre *Hrh1*^f/f^ mice (Fig. 3C). Although a few attacks by CaMKII-Cre *Hrh1*^f/f^ mice were observed, the number of attacks was not statistically significant compared to that of control mice. Taken together, data from the resident-intruder test indicated that astrocyte-specific H_1_ receptor knockout mice were highly aggressive and suggest a role of H_1_ receptors on astrocytes in the regulation of aggressive behaviour.

### Deletion of astrocytic H_1_ receptors delays the onset of nocturnal activity

**Figure 4:**
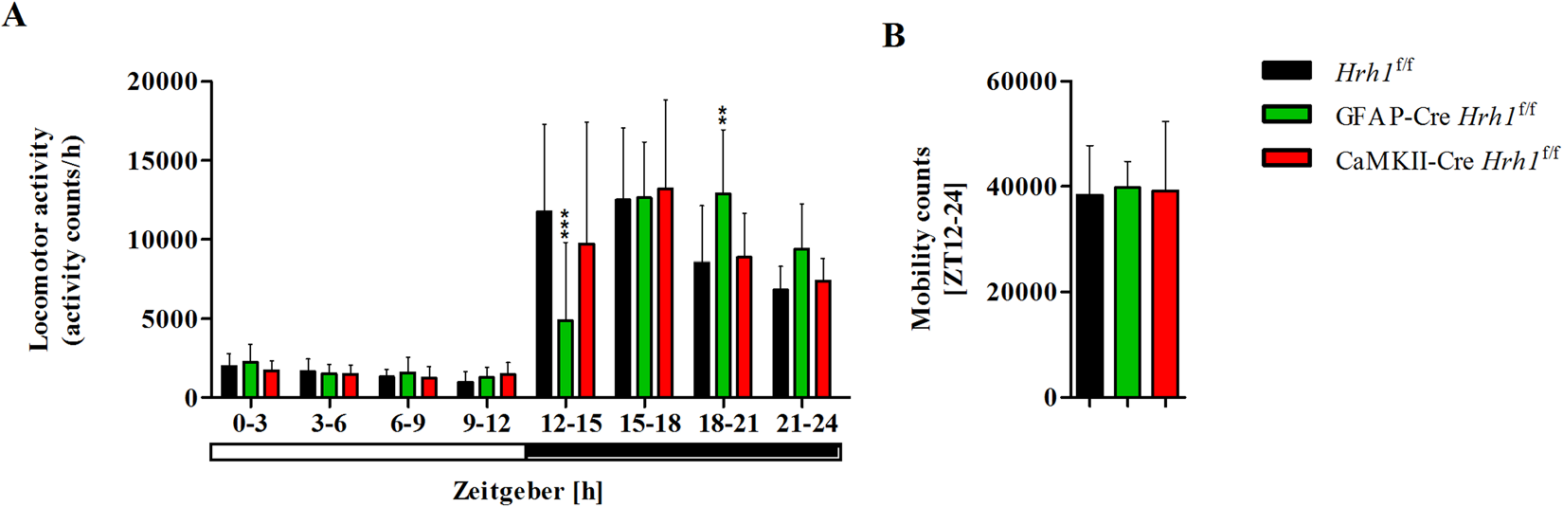
Deletion of astrocytic H_1_ receptors affected the onset of nocturnal activity. Total home cage locomotor activity (**A**) and activity during the dark period (**B**) in the home cages of cKO mice and controls. (**A**) Mice were placed in individual home cages, and their vigilance level was assessed by an infrared detector for 48 h. Mobility counts were averaged for 3-hour intervals (n = 9–10). Data were assessed with two-way ANOVA and Bonferroni post-hoc test (**p < 0.01 and ***p < 0.001). (**B**) Mobility counts for the entire dark period (ZT 12–24) (n = 9–10). Data were analysed with one-way ANOVA and Tukey’s post-hoc test.

The histaminergic system is closely associated with wakefulness and sleep and H_1_ receptors were shown to affect quantity and quality of wakefulness^12,31,32^. Deletion of H_1_ receptors could potentially reflect on home cage locomotor activity of *Hrh1* cKO mice. Continuous monitoring of mice in their home cages during the light and dark phases revealed that GFAP-Cre *Hrh1*^f/f^ mice exhibited obvious changes in locomotor activity when compared to *Hrh1*^f/f^ controls (Fig. 4). All groups exhibited very low activity during the light phase. However, while the level of vigilance typically rises quickly after onset of the dark phase and peaks within a few hours, GFAP-Cre *Hrh1*^f/f^ mice demonstrated delayed onset of nocturnal activity and were highly active at ZT 18–21 (Fig. 4A). In comparison, the activity level of controls peaked within the first 3 hours of the dark phase (ZT 12–15). The activity of CaMKII-Cre *Hrh1*^f/f^ mice followed the same pattern as that of the control group. Significantly decreased activity of GFAP-Cre *Hrh1*^f/f^ mice at ZT 12–15 and increased activity at ZT 18–21 did not impact on their overall activity during the dark phase (Fig. 4B).

Taken together, the obtained results showed that astrocytic H_1_ receptors are important for high vigilance during the early dark phase.

### Qualitative changes in wakefulness in GFAP-Cre *Hrh1*^f/f^ mice

**Figure 5:**
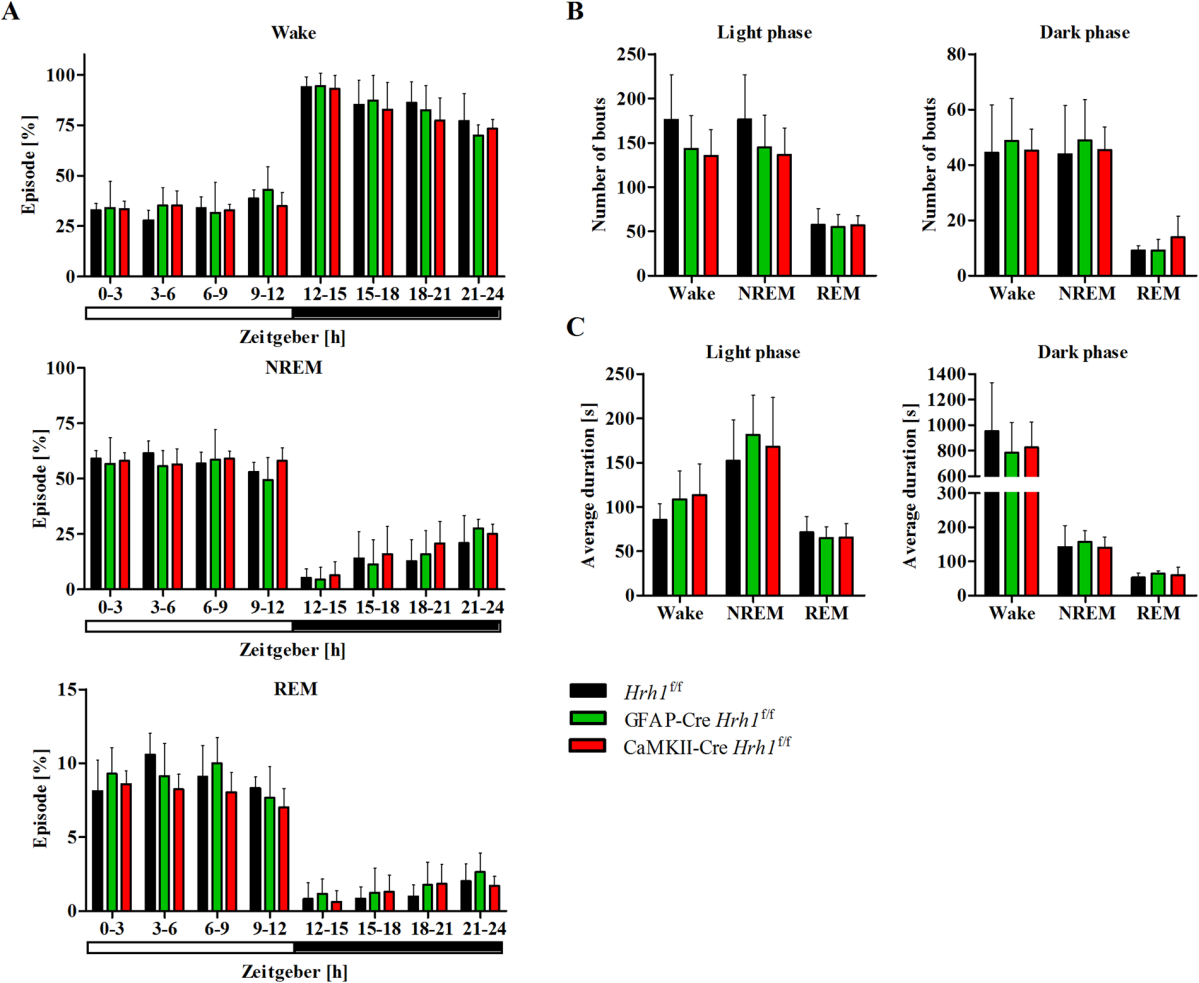
Sleep-wake cycle was not affected by conditional *Hrh1* deficiency. Results from 24 h EEG/EMG recordings of cKO and control mice. (**A**) Time spent in each stage averaged for 3-hour intervals (n = 6–7). (**B**) Graphs represent the number of bouts for each stage during the light (left) and dark (right) phase (n = 6–7). (**C**) Graphs represent the average duration of each stage during the light (left) and dark (right) phase (n = 6–7). All data were analysed with one-way ANOVA and Tukey’s post-hoc test.

**Figure 6:**
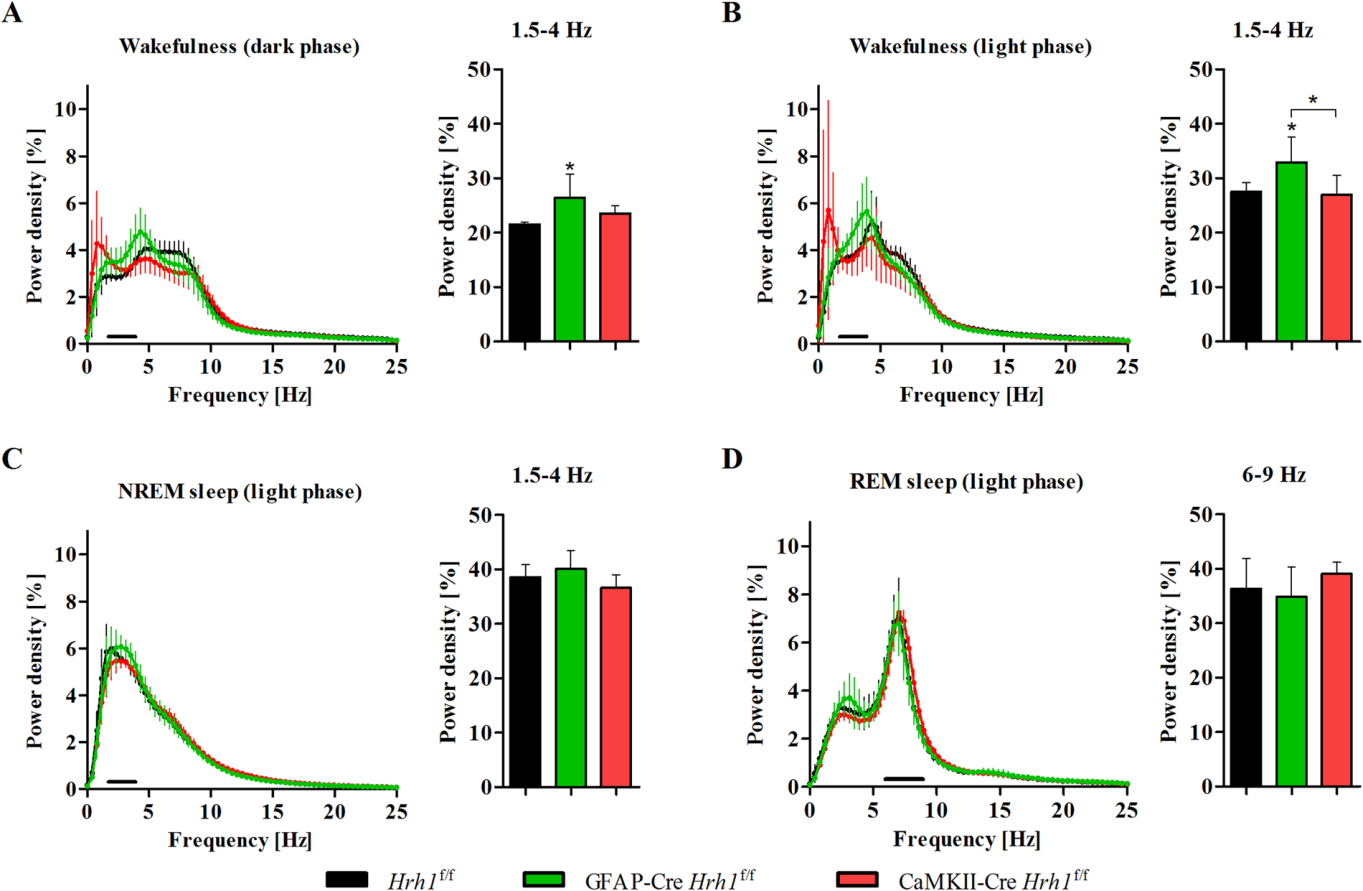
Astrocytic H_1_ receptor deficiency was associated with an increased amount of delta waves during wakefulness Spectral distribution of EEG power densities was analysed for stages scored as wake (**A**,**B**), NREM sleep (**C**), and REM sleep (**D**). Left graphs: The power spectra were analysed for 0–25 Hz of each vigilance state over a period of 12 h. Right graphs: The percentage of delta waves (1.5–4 Hz) across the groups was assessed for wakefulness (dark and light phase) and NREM sleep (light phase). The percentage of theta waves (6–9 Hz) was analysed for REM sleep (light phase) (n = 6). Statistical differences were assessed with one-way ANOVA and Tukey’s post-hoc test (*p < 0.05).

Next, we recorded electroencephalograms (EEGs) and electromyograms (EMGs) to investigate whether changes in circadian activity were associated with disturbances in the sleep-wake cycle. The sleep-wake cycle was similar across all groups. cKO and control mice spent the same percentage of time in each stage in all 3 h intervals: wakefulness, non-rapid eye movement (NREM) sleep, and rapid eye movement (REM) sleep (Fig. 5A). The number of bouts and average duration of each stage during light and dark phases were unchanged in cKO mice (Fig. 5B,C). Data from EEG power spectra showed the distribution of frequencies during wakefulness, NREM sleep, and REM sleep (Fig. 6). The power density of delta waves at 1.5–4 Hz during wakefulness in the day and at night was significantly increased in GFAP-Cre *Hrh1*^f/f^ mice (Fig. 6A,B). Furthermore, we analysed the power density at low frequencies in the range of 0.5–1 Hz during wakefulness, but did not find a significant difference among the groups. Spectral power densities during NREM and REM sleep were similar across all groups (Fig. 6C,D).

Taken together, these findings indicate that the quantity and quality of sleep were not affected by conditional *Hrh1* deletion; however, astrocytic *Hrh1* deficiency had a significant impact on the prominence of delta waves at frequencies of 1.5–4 Hz during wakefulness.

## Discussion

Histamine H_1_ receptors in the CNS are involved in various physiological processes. To date, it remains unknown how neuronal and astrocytic H_1_ receptors contribute to different aspects of higher brain function. In this study, we observed that both neuronal and astrocytic H_1_ receptors regulated anxiety-like behaviour. Astrocyte-specific *Hrh1* knockout mice showed increased aggressive behaviour, altered circadian rhythm, and changes in quality of wakefulness. Finally, neuronal H_1_ receptors were important for recognition memory.

In this study, we developed novel cKO mice with selective *Hrh1* deletion from either astrocytes or neurons, providing a sophisticated tool to study behaviour *in vivo*. *Hrh1* cKO mice showed significantly decreased *Hrh1* expression in astrocytes and neurons. Results from immunohistochemical stainings revealed increased Cre expression in brain sections of CaMKII-Cre mice than in brain sections of GFAP-Cre mice. The increased Cre expression in CaMKII-Cre mice may account for the lower *Hrh1* expression in neuron-specific KO mice compared to that in astrocyte-specific KO mice. Moreover, previous research showed that GFAP-Cre transgenic mouse line 77.6 exhibited “scattered mosaic pattern”-like Cre expression^29^. This result may explain why *Hrh1* expression was only reduced by half. Future studies will have to investigate whether further reduction of astrocytic *Hrh1* expression results in a stronger or distinct phenotype. *Hrh1* expression was generally lower in astrocytes than in neurons, suggesting that neuronal H_1_ receptors comprise majority of H_1_ receptors in the brain. Deletion of H_1_ receptors from astrocytes did not affect histamine concentrations in various brain regions of GFAP-Cre *Hrh1*^f/f^ mice. In contrast, hypothalamic histamine concentration was twice as high in CaMKII-Cre *Hrh1*^f/f^ mice than in controls. Histamine is produced in histaminergic neurons originating in the hypothalamic TMN; it is possible that this may be a compensatory mechanism for impaired H_1_ receptor signaling. However, under baseline conditions, histamine release and the concentration of its metabolite 1-methylhistamine are strongly correlated. Therefore, the stable concentration of 1-methylhistamine suggests that histamine release remained unchanged. Dopamine is metabolised to two intermediates, DOPAC and 3-methoxytyramine (3-MT), that are subsequently converted into homovanillic acid (HVA). DOPAC concentrations were increased in two brain regions of CaMKII-Cre mice, but the concentration of 3-MT was not affected. Since the sum of DOPAC, 3-MT, and HVA, which represents the overall metabolism of dopamine, was not altered among the study groups, changes in DOPAC level did not affect the aspects of mouse behaviour that were examined.

We assessed behaviour that is reportedly linked to H_1_ receptor signalling, including exploratory and anxiety-like behaviour, memory, learning, aggression, and circadian rhythms. Exploratory behaviour of cKO mice was not affected in a novel environment. Although several researchers have reported the involvement of H_1_ receptors in exploratory behaviour and found it to be decreased in conventional *Hrh1* KO mice^7,8^, our data did not support these observations. The memory tasks conducted indicated that memory function is complex and involves different cell signaling pathways in the brain. Unlike in a previous study that used conventional *Hrh1* mice^33^, the spatial memory function was not affected by conditional *Hrh1* deletion. However, CaMKII-Cre *Hrh1*^f/f^ mice showed impaired memory function during the novel object recognition test. Mice with astrocytic *Hrh1* deficiency did not display any changes. H_1_ receptors are known to be important for memory and long-term potentiation in the hippocampus^34,35^, but the role of H_1_ receptors on astrocytes in memory has not been established. To the best of our knowledge, these results show for the first time that neuronal, but not astrocytic, H_1_ receptors are important for recognition memory. Further, we observed that anxiety-like behaviour was significantly decreased in both cKO mouse models. In agreement with our findings, an *Hrh1* knockout study and a pharmacological study using the H_1_ receptor antagonist diphenhydramine reported that H_1_ receptor signalling mediated anxiety-like behaviour^8,36^. Additionally, our results indicated that both neuronal and astrocytic H_1_ receptors played a crucial role in anxiety. Our data suggest that both cell types can independently regulate anxiety-like behaviour, but further studies are required to validate the roles of neuronal and astrocytic H_1_ receptors in this process.

The debate on how histamine modulates aggression continues to this day^8,36–38^. GFAP-Cre *Hrh1*^f/f^ mice showed significantly increased aggression towards intruders. The present study reports, for the first time, that H_1_ receptors on astrocytes regulate aggressive behaviour. In an earlier study with conventional *Hrh1* KO mice, resident mice were significantly less aggressive compared to controls^8^. However, a noticeable difference in the period of individual housing among the previous and the present study, six months versus one weeks, has to be considered when comparing the findings. Other publications by Ray *et al*. and Nath *et al*. reported that treatment with H_1_ receptor antagonists increased, while H_2_ receptor antagonists decreased, foot shock-induced aggressive behaviour^38,39^. The authors concluded that H_1_ and H_2_ receptors possess interlinked signalling that can alter aggressive behaviour. The hypothesis regarding the interlinked nature of H_1_ and H_2_ receptor signalling was further explored by Alonso *et al*., who revealed active cross-talk and co-trafficking of these two histamine receptors^40^. As astrocytes express both H_1_ and H_2_ receptors, the question arises whether these receptors have opposing roles and counteract each other in the context of aggression. Our results emphasise the role of H_1_ receptors on astrocytes in aggressive behaviour. Nevertheless, further research is required to fully understand the role of astrocytic H_1_ receptor signalling and its potential link to H_2_ receptor signalling in the context of aggressive behaviour.

Home cage locomotor recordings showed a delayed onset of nocturnal activity in GFAP-Cre *Hrh1*^f/f^ mice. The importance of H_1_ receptor activation in arousal and wakefulness is well-established^12,32,41^, and our data support the concept of astrocytic H_1_ receptors as a key player in this process. We conjectured that changes in the level of vigilance may be associated with sleep-wake cycle disturbances; however, EEG/EMG recordings did not indicate any quantitative changes in sleep behaviour, relative time spent in each sleep stage, number of sleep bouts, or average duration of episodes in different sleep stages. Moreover, EEG/EMG recordings revealed that mice were awake after the onset of darkness, similar to controls. These results emphasize that H_1_ receptors on astrocytes did not regulate the quantity of sleep, and the observed changes in circadian rhythm were a result of reduced locomotor activity in GFAP-Cre *Hrh1*^f/f^ mice. Conventional *Hrh1* KO mice showed altered sleep behaviour, with decreased time spent in the wake stage during the early dark phase^32^, but none of the cKO mouse models exhibited this phenotype. There are different scenarios that could explain why the observed phenotype is different from the one previously observed. First, to date, the role of H_1_ receptors has been studied with conventional KO mice, which may have had additional impact on the phenotype in previous studies. Conventional KO mice are characterised by non-selective *Hrh1* KO in all *Hrh1*-expressing cells at all times. In contrast, Cre-dependent cKO mice still express the gene of interest until Cre expression ensures cell-specific recombination at the *loxP* sites and subsequent gene deletion. Therefore, we cannot rule out the possibility that previous observations were associated with these differences between the two mouse models. Second, it is possible that H_1_ receptors on astrocytes and neurons jointly regulate sleep behaviour, and conditional gene deletion is insufficient to cause significant changes. It will thus be critical to examine whether double cKO mice, CaMKII-GFAP-Cre *Hrh1*^f/f^, could mimic the phenotype of conventional *Hrh1* KO mice. Third, we previously acknowledged the low and “scattered mosaic pattern”-like GFAP-dependent Cre expression. It might be possible that further reduction of *Hrh1* expression in astrocytes is required to induce a stronger phenotype, possibly comparable to that of the conventional KO mouse.

To further explore the reasons for reduced vigilance, we investigated the quality of wakefulness and sleep in cKO mice by analysing EEG power spectra of all groups. Although the quality of NREM and REM sleep remained unchanged, astrocyte-specific cKO mice revealed an abnormal increase in delta waves at 1.5–4 Hz during wakefulness. An increase in delta waves during wakefulness has been described by Parmentier *et al*. in KO mice lacking the histamine-synthesising enzyme histidine decarboxylase^42^. Delta waves are a major indication for insufficient cortical activation and are associated with a drowsy state and low vigilance during wakefulness. Moreover, histamine perfusion significantly decreases the amount of delta waves during wakefulness^43^. We hypothesise that deletion of H_1_ receptors from astrocytes prevented activation of signalling cascades, which usually ensure sufficient cortical activity after awakening to maintain high vigilance. H_1_ receptor-mediated signalling has the ability to adequately stimulate cortical activity through the activation of cholinergic and serotonergic neurons^2^. It is possible that *Hrh1*-expressing astrocytes play a critical role in exciting these neurons through the release of bioactive molecules. Concluding from the obtained results, we propose that the histaminergic system is crucial to maintain wakefulness with high vigilance after awakening, and that this signalling is mediated through the activation of H_1_ receptors on astrocytes. However, deletion of *Hrh1* in astrocytes did not affect the normal sleep-wake cycle, quantity, or quality of sleep, because neither sleep latencies nor the duration of sleep episodes were affected. Wakefulness has typically been associated with H_1_ receptors, but previous studies did not confirm distinct roles of neurons and astrocytes in this process. This is the first report indicating that H_1_ receptors on astrocytes are important for the maintenance of high vigilance after awakening. Given the incomplete deletion of *Hrh1* in astrocytes, it is possible that we are currently observing a phenotype that can somewhat cope with the partial gene deletion resulting in impaired wakefulness, but not disturbed sleep behaviour. It will be of great interest to study spontaneous locomotor activity, sleep, and wakefulness in a mouse model that can achieve complete *Hrh1* deletion in astrocytes.

This study provided new functional insights regarding the role of H_1_ receptors on neurons and astrocytes, and novel *Hrh1* cKO mice were highlighted as a powerful tool to study cell-specific functionality. Nevertheless, several limitations of this study need to be addressed by future research. First, we did not provide data showing the distribution of neuronal and astrocytic H_1_ receptors in different brain regions. Currently, there are no good antibodies against the H_1_ receptor, and we were unable to design a probe with high sensitivity and specificity for *in situ* hybridization purposes. Second, this study did not investigate the roles of brain regions implicated in the observed phenotypic changes. Adeno-associated virus (AAV)-mediated delivery of Cre-recombinase can selectively achieve genetic engineering in targeted cell populations and represents an advanced strategy to examine brain circuits involved in H_1_ receptor-mediated behaviour in the future. For instance, AAV-mediated regulation of neuronal-specific Cre recombinase expression in the hippocampus will enable investigation of the roles of hippocampal H_1_ receptors in memory function. Third, future research should examine the relationship between observed behavioural changes and their underlying molecular mechanisms, as we did not investigate the underpinning cellular changes in cKO mice. It is well established that astrocytes respond to H_1_ receptor activation in various ways, including glutamate homeostasis, energy metabolism and inflammatory processes. Thus, it will be important to study molecular changes and their consequences in the cKO mouse, because an interpretation of the observed phenotype can only be speculative at this moment.

Finally, astrocyte-specific KO mouse models that will achieve better *Hrh1* deletion have to confirm our observations as current negative results, especially the results that are opposing to those in studies with conventional KO mice, might be a result of incomplete Cre recombination in astrocytes.

In conclusion, our study stresses the importance of H_1_ receptors on neurons and astrocytes in distinct physiological processes. Our findings contribute to our understanding of how H_1_ receptors on different cell types are involved in the development of pathological states and highlight astrocytes as a novel therapeutic target.

## Methods

### Animals

Mice were kept on a 12 h light-dark cycle (ZT 0–12: lights on; ZT 12–24: lights off) with controlled humidity and temperature. Unless otherwise described, mice were housed in groups with up to five animals per cage with *ad libitum* access to a commercial standard diet (Labo MR stock; Nosan Corporation, Yokohama, Japan) and water.

The care and use of animals in this study was conducted in accordance with the Principles for the Care and Use of Research Animals of Tohoku University, Sendai, Japan. All animals and gene-recombination experiments were given ethical approval from the Tohoku University Center for Laboratory Research and Tohoku University Center for Gene Research, respectively.

### Generation of cKO mice

*Hrh1*^f/f^ mice were designed by UNITECH Co. Ltd (Chiba, Japan). Briefly, a targeting vector was used to introduce two *loxP* sites into exon 3 of the allele of interest, flanking the coding sequence of *Hrh1* (Fig. 1A). GFAP-Cre (B6.Cg-Tg(Gfap-cre)77.6Mvs/2J) mice were purchased from Jackson Laboratory (Bar Harbor, ME, USA) and CaMKII-Cre (C57BL/6-TgN(a-CaMKII-nlCre)/10) mice from RIKEN BioResource Center (BRC) (Tsukuba, Japan). Generation of GFAP-Cre *Hrh1*^f/f^ and CaMKII-Cre *Hrh1*^f/f^ mice, and Cre-recombination, was achieved by crossing *Hrh1*^f/f^ mice with GFAP-Cre and CaMKII-Cre mice, respectively. Genotypes of offspring and successful recombination were determined by genomic DNA PCR. Cell-specific deletion of *Hrh1* was confirmed with real-time PCR.

### Primary cell cultures

Primary astrocytes and neurons were isolated at P0–P1 as previously described with some modifications^44^. Brains were removed and kept in Hank’s balanced salt solution containing 100 IU/mL benzylpenicillin potassium and 100 μg/mL streptomycin sulphate until the meninges were removed. The tissue was homogenized and incubated with 0.25% trypsin (Nacalai Tesque, Kyoto, Japan) and 0.01% DNase I (Roche Applied Science, Penzberg, Germany) for 10 min at 37 °C. The enzymes were inactivated with horse serum (GE Healthcare Life Sciences, Malborough, MA, USA), following which, the cells were pelleted. Finally, cells for astrocyte cell culture were resuspended in Dulbecco’s modified Eagle medium (DMEM)/nutrient mixture F12 (Life Technologies, Carlsbad, CA, USA) supplemented with 10% foetal bovine serum (GE Healthcare Life Sciences), 100 µM non-essential amino acids (Life Technologies), 100 IU/mL benzylpenicillin potassium, and 100 μg/mL streptomycin sulphate. Cells for neuronal cell culture were resuspended in Neurobasal medium (Life Technologies) containing B-27 supplement (Life Technologies) and 0.5 mM l-Alanyl-l-Glutamine (Wako Pure Chemical Industries, Ltd., Osaka, Japan). Resuspended cells were passed through a 100 µm cell strainer (Greiner, Kremsmünster, Austria), and plated on poly-l-lysine (Sigma-Aldrich, St. Louis, MO, USA) coated 6-well plates. The cells were cultured under a humidified atmosphere of 5% (neurons) or 10% (astrocytes) CO_2_ at 37 °C. The medium was changed every 3 days until cells were used for further studies.

### Isolation of genomic DNA

Genomic DNA from mouse tail-cut samples was obtained as described by Truett *et al*. with minor modifications^45^. The tissue was lysed with 50 mM NaOH at 95 °C for 30 min and subsequent centrifugation. The supernatant was subjected to PCR analysis.

DNA from brain homogenates of mice aged 8–12 weeks old or from primary cell cultures was isolated using the phenol/chloroform method. Cells were lysed and homogenised before chloroform was added. After phase separation, the aqueous phase was used to perform ethanol precipitation and purify the genomic DNA. The dissolved DNA was used for PCR analysis.

### Isolation of total RNA

Total RNA from brain homogenates of mice aged 8–12 weeks was isolated using RNAiso Plus (Takara Bio Inc., Ohtsu, Japan) according to the manufacturer’s protocol. Total RNA from primary cell cultures was isolated using NucleoSpin® RNA Plus (Macherey-Nagel GmbH & Co. KG, Düren, Germany) according to the manufacturer’s instructions.

### Conventional PCR

Conventional PCR was run to confirm the presence of the *Hrh1 loxP* sites, Cre transgene, and successful recombination. Genomic DNA and specific primers (Table 2) were used with Toyobo KOD FX neo (Toyobo, Osaka, Japan) according to the manufacturer’s protocol.

**Table 2:** Primer sequences for genotyping transgenic mice.

Genotype	Primer name	Nucleotide sequence (5′ → 3′)	Product size (bp)	Reference
CaMKII-Cre	CaMKpro-F Cre	ACGGGAACAGGGCGTTTCGGAGGTGGTTGC CTAATCGCCATCTTCCAGCAGG	1300	Riken BRC
	mLC3ex3GT mLC3ex4AG	TGAGCGAGCTCATCAAGATAATCAGGT GTTAGCATTGAGCTGCAAGCGCCGTCT	500	Mizushima *et al*.^53^
GFAP-Cre	oIMR1084 oIMR1085	GCGGTCTGGCAGTAAAAACTATC GTGAAACAGCATTGCTGTCACTT	100	Jackson Laboratory
	oIMR7338 oIMR7339	CTAGGCCACAGAATTGAAAGATCT GTAGGTGGAAATTCTAGCATCATCC	324	Jackson Laboratory
*Hrh1* Flox	*loxP* insert	AACAACAATTCTTGGGCAGTG TGATGCTAGTTTGGAGGTAGTTAGG	157/329	This study
	Recom- bination	GCCGCAAAACAATTGATAACTTC CTTTTTGGAGAAGGCGAGTG	3244/1106	This study

### Real-time PCR

Previously isolated RNA from whole mouse brain or primary astrocytes was reverse transcribed with PrimeScript RT Master Mix (Toyobo), according to the manufacturer’s protocol. cDNA was run on a two-step PCR using TB Green™ *Premix Ex Taq*™ (Toyobo). The cycling conditions were as follows: pre-denaturation at 95 °C for 30 s, followed by 40 cycles of denaturation at 95 °C for 5 s and extension at 60 °C for 30 s. Specific primers for the genes of *Hrh1* (5′ → 3′ sense: TCACTCCAGGCCTCACATG; antisense: CAAAGTTCTCATCCCAAGTTTCCA, 95 bp), Cre recombinase (*Cre*) (5′ → 3′ sense: CGACCAGGTTCGTTCACTCA; antisense: CAGCGTTTTCGTTCTGCCAA, 186 bp), and glyceraldehyde 3-phosphate dehydrogenase (*Gapdh*) (5′ → 3′ sense: AGAACATCATCCCTGCATCC; antisense: CACATTGGGGTAGGAACAC, 91 bp) were used for amplification.

### Behavioural analysis

Male mice aged 8–12 weeks positively genotyped for one of the three experimental groups were assessed in a series of behavioural tests for exploratory behaviour, anxiety, learning, memory, circadian activity, and aggression. All behavioural experiments were performed during the dark period. During the open field, light-dark box, elevated plus-maze, Y-maze, and locomotor activity tests, the movement tracking software automatically assessed the behaviour of the mice. In the resident-intruder and novel object recognition tests, all obtained data were analysed by two different investigators, who were blinded to the mice’ genotypes. We avoided to keep mice in isolated environment except for resident-intruder test and home cage locomotor activity test.

### Open field test

The open field test was performed in an open arena of 50 × 50-cm (Muromachi Kikai Co., Ltd., Tokyo, Japan) for 30 min at 40 lux^46^. The arena was surrounded by Plexiglas containing a photo-beam apparatus with 64 photo cells (BTA-1®, Muromachi). There were eight photocells on each side that tracked movements. The two outer photo cells on each side (12.5 cm from any wall) were considered the periphery, while the inner part of the arena was considered the centre.

### Y-maze spontaneous alternation test

Spatial working memory of cKO mice was assessed using the Y-maze test^47^. The arms of the maze were 40 cm long, 8 cm high, and 3 cm wide at the bottom, and converged at an equal angle. Mice were placed at the end of one of the arms and were allowed to move freely in the maze. All movements were recorded using SMART® software (Harvard Apparatus, Holliston, MA, USA). Each session lasted for 8 minutes and was performed at 100 lux. An alternation was defined as entries into all three arms on consecutive choices.

### Novel object recognition

The novel object recognition test was used to assess working memory of cKO mice^48^. The test was carried out in a transparent Plexiglas box (30 × 30 × 30 cm) at 40 lux. The test was performed for 3 days with one session per day. On day 1, mice were habituated and were allowed to freely explore the novel environment without any objects for 10 min. On the next day, mice were familiarised with two identical objects for 5 min. The objects were fixed at two positions with the same distance to the walls. After 24 hours, a test trial was conducted. During the test trial, one of the familiar objects was replaced with an unknown object; mice were allowed to freely explore the environment for 5 min. The objects were made in-house from ordinary laboratory equipment; 75 cm² cell culture flasks filled with water and 50 mL black falcon tubes were used. Both objects were approximately 9 cm high, made from plastic, and featured different colours and shapes. The exploration time reflects the total time spent at both objects together; the cut-off time for total exploration was 20 s. The preference ratio indicates the time spent at the new object relative to the total exploration time. The statistical difference of the preference ratio during training and test session for control mice was confirmed with Student’s *t*-test. Changes in the preference ratio among the experimental groups during the test session were assessed using two-way ANOVA and Bonferroni post-hoc test.

### Light-dark box test

The light-dark box test was performed in a box featuring an illuminated (>400 lux) and a dark (<10 lux) compartment with a small partition, which allowed mice to move between the two compartments^49^. Mice were placed in the dark compartment, and the time spent in each compartment was recorded over 10 min with Opto-Max® software (Columbus Instrument, Columbus, OH, USA).

### Elevated plus-maze test

The elevated plus-maze test was conducted as previously described with minor modifications^50^. The maze consisted of two open arms and two closed arms with walls on the side and at the end, and a central platform (EPM-04®; Muromachi). The maze was elevated 40 cm from the floor. The light intensity at the central platform was 100 lux. Mice were placed at the end of a closed arm and were allowed to explore the maze for 10 min. SMART® software (Harvard Apparatus) was used to record movements.

### Home cage locomotor activity

Home cage locomotor activity was assessed as previously described^51^. Mice in individual home cages were habituated for 24 hours prior to the recording of locomotor activity. Locomotor activity was measured by an epoch-making activity monitoring system that uses an infrared beam to sense the animal’s movements (SUPERMEX, Muromachi). Data were collected and analysed by the CompACT AMS® software (Muromachi). The activity counts were summarised in 5 min epochs and recorded continuously for 48 h.

### Resident-intruder test

The resident-intruder test was performed as previously described^52^. Mice (residents) were housed individually for 1 week prior to the experimental day. Testing was performed in the home cage of the resident. The intruder, an unfamiliar male mouse, was placed directly into the home cage of the resident for 5 minutes. The mice were allowed to move freely and interact during the test. The test was recorded and subsequently, the attack latency, number of attacks, and cumulative attack time were scored.

### Measurement of histamine and 1-methylhistamine

Brain regions of interest were isolated from perfused mice and snap frozen in liquid nitrogen. Each brain region was homogenised in 0.4M perchloric acid. Brain homogenates were centrifuged at 15,000 × *g* for 15 min at 4 °C. Subsequently, the supernatants were transferred to new tubes and centrifuged again as described. The supernatants were transferred before applying the samples to an HPLC system for measurement of histamine and 1-methylhistamine concentrations. Samples were separated on an SC-5ODS column (3.0 mm i.d. × 150 mm; Eicom, Kyoto, Japan) with 0.1 M sodium acetate (pH 4.6)-methanol (91:9, v/v) mobile phase that was supplemented with 220 mg/L sodium 1-octanesulfonate at a flowrate of 500 µL/min at 40 °C. Samples were mixed with 80 mg/L OPA solution containing 2% methanol and 0.004% 2-mercaptoethanol (flowrate: 100 µL/mL), and 0.5 M potassium carbonate solution (flowrate: 100 µL/mL). Histamine and 1-methylhistamine were excited at 335 nm and measured at 450 nm with a fluorescent detector (Hitachi, Tokyo, Japan).

### Sleep monitoring

For EEG and EMG recordings, mice were implanted with a head mount (Pinnacle Technology Inc., Laurence, KS, USA) parallel to the sagittal suture. Four stainless steel screws were placed 1.5 mm lateral to the sagittal suture, 2.0 mm anterior of bregma, and 4.0 mm posterior to bregma to record EEG signals. EMG signals were acquired by a pair of multi-stranded stainless steel wires inserted into the neck extensor muscles. One week after surgery, animals were habituated for 3 days before EEG/EMG signals were acquired with a 3-channel EEG/EMG tethered system (Pinnacle Technology Inc.) and digitalised by SIRENIA SLEEP PRO^®^ software (Pinnacle Technology). EEG and EMG data were recorded in 10 s epochs and automatically scored by Sleep Sign^®^ software (Kissei Comtec, Matsumoto, Japan). Automatically scored data were manually inspected and corrected if required.

### EEG power spectral analysis

EEG spectral power was calculated for 10 s epochs in 0.4 Hz bins using fast Fourier transformation with Sleep Sign^®^ software (Kissei Comtec). Power spectra were analysed for frequencies 0–25 Hz for each vigilance state in 12-hour intervals (dark and light phases). Data were standardised for each animal by summing up the power of all 0.4 Hz bins across one vigilance state (total power) before determining the percentage of selected frequency ranges. Frequencies selected for analysing were 1.5–4 Hz for delta waves and 6–9 Hz for theta waves. Power spectral densities at different frequency ranges were expressed as percentage relative to the total power.

### Statistical analysis

All statistical analyses were performed with Prism 5 software (GraphPad, La Jolla, CA, USA). Significant differences were assessed by one-way ANOVA with Tukey’s post-hoc test, two-way ANOVA with Bonferroni post-hoc test, or Student’s *t* tests; *p* < 0.05 was the threshold for statistical significance. Results are expressed as the mean ± standard deviation.

## Supplementary information

Supplementary information
