# Supplementary material for: Histamine H1 receptor on astrocytes and neurons controls distinct aspects of mouse behaviour
Source: Sci Rep. 2019 Nov 11;9:16451. doi: 10.1038/s41598-019-52623-6 (PMC6848115; doi:10.1038/s41598-019-52623-6)
Supplement: Supplementary file 1 — Supplementary information [file 41598_2019_52623_MOESM1_ESM.pdf]

Supplementary information

Histamine H_1 receptor on astrocytes and neurons controls distinct aspects of mouse behaviour

Anikó Kárpáti[a], Takeo Yoshikawa[a]*, Fumito Naganuma[a,b], Takuro Matsuzawa[a], Haruna Kitano[a], Yo Yamada[a], Mariko Yokoyama[a], Akira Futatsugi[c], Katsuhiko Mikoshiba[d] and Kazuhiko Yanai[a]

[a]Department of Pharmacology, Tohoku University Graduate School of Medicine, 2-1 Seiryo-machi, Aoba-ku, Sendai, 980-8575 Japan

[b]Division of Pharmacology, Faculty of Medicine, Tohoku Medical and Pharmaceutical University, 1-15-1 Fukumuro, Miyagino-ku, Sendai, 983-8536 Japan

[c]Department of Basic Medical Sciences, Kobe City College of Nursing, 3-4 Gakuen-nishi-machi, Nishi-ku, Kobe, 651-2103 Japan

[d]Shanghai Institute for Advanced Immunochemical Studies, ShanghaiTech University, Shanghai, 201210 China

Supplementary methods

Immunohistochemistry

Brain tissues were perfused with 4%-paraformaldehyde phosphate buffer solution (Nacalai Tesque, Kyoto, Japan) and were kept in 30% sucrose until equilibration. Brain tissues were then embedded into Tissue-Tek® O.C.T. Compound (Sakura Finetek USA Inc., Torrance, CA, USA) and cryosectioned into sections of 50-μm thickness before being mounted onto glass slides. Mounted sections were permeabilised and blocked before incubation with primary antibodies (rabbit polyclonal anti-GFAP [Z0334; Dako, Glostrup, Denmark; 1:500] or rabbit monoclonal anti-NeuN [ab177487; Abcam, Cambridge, UK; 1:1,100] and mouse monoclonal anti-Cre-recombinase [MAB3120; Millipore, Burlington, MA, USA; 1:1,000]) overnight at 4 °C. The next day, sections were washed and incubated with secondary antibodies (anti-mouse IgG Alexa Fluor488 [A11017; 1:1,000] and anti-rabbit IgG Alexa Fluor568 [A11036; 1:1,000], both purchased from Invitrogen [Carlsbad, CA, USA]) before sections were mounted using VECTASHIELD® Hardset™ Antifade Mounting Medium with DAPI (Vector Laboratories, Burlingame, CA, USA). Images were obtained with a Nikon C2si confocal microscope and digitalised with NIS-Elements Imaging Software (Nikon, Tokyo, Japan).

Measurement of monoamines and monoamine metabolites

Brain regions of interest were isolated from perfused mice and snap frozen in liquid nitrogen. Each brain region was homogenised in 0.4 M perchloric acid. Brain homogenates were centrifuged at 15,000 × *g* for 15 min at 4 °C. Subsequently, the supernatants were transferred to new tubes and centrifuged again as described. The supernatants were transferred before applying the samples to an HPLC system for measurement of monoamine neurotransmitters and monoamine metabolites. Sample separation was performed at 25 ºC on an SC-5ODS column (2.1 mm i.d. × 150 mm; Eicom) in the presence of 0.1 M citric acid/0.1 M acetic acid

(pH 3.9)-methanol (83:17, v/v) buffer containing 140 mg/L sodium 1-octanesulfonate and 5 mg/L EDTA/2Na with a flow rate of 500 μL/min. Monoamine neurotransmitters (dopamine, norepinephrine, and serotonin) and their metabolites (3,4-dihydroxyphenylacetic acid [DOPAC], 3-methoxytyramine, homovanillic acid and 5-hydroxyindoleacetic acid) were measured with an HTEC-500 electrochemical detection system (Eicom).

Real-time PCR

Total RNA from tissue homogenates were isolated using RNAiso Plus (Takara Bio Inc., Ohtsu, Japan) according to the manufacturer's protocol. Reverse transcribed samples were amplified with KOD FX neo (Toyobo, Osaka, Japan) using oligonucleotide primers for glyceraldehyde 3-phosphate dehydrogenase (*Gapdh*) (PCR product size 477 bp) and histamine H_1 receptor (*Hrh1*) (PCR product size 523 bp) as described previously (Iida et al. *Glia*. 2015;63(7):1213-25).

Supplementary Figure S1

Figure 1B
CaMKII-Cre transgene

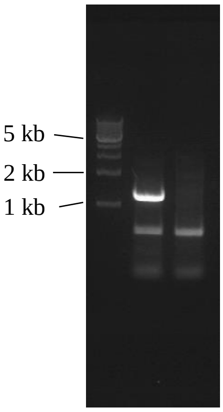

GFAP-Cre transgene

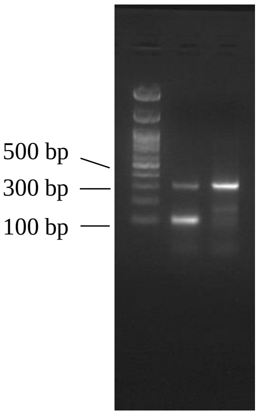

Figure 1D
Recombination primary cell cultures

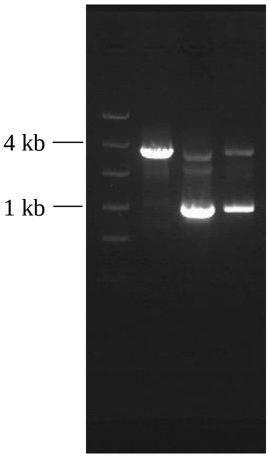

Figure 1E
Recombination whole brain homogenate

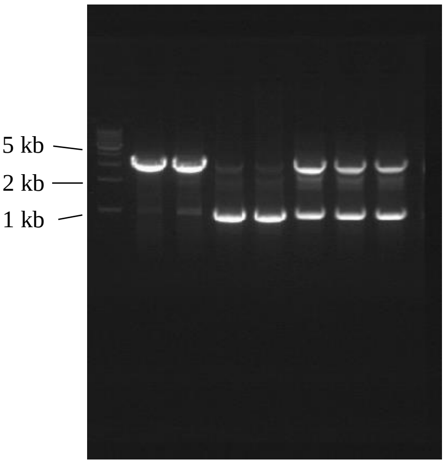

Original full length agarose gels of main manuscript

Supplementary Figure S2

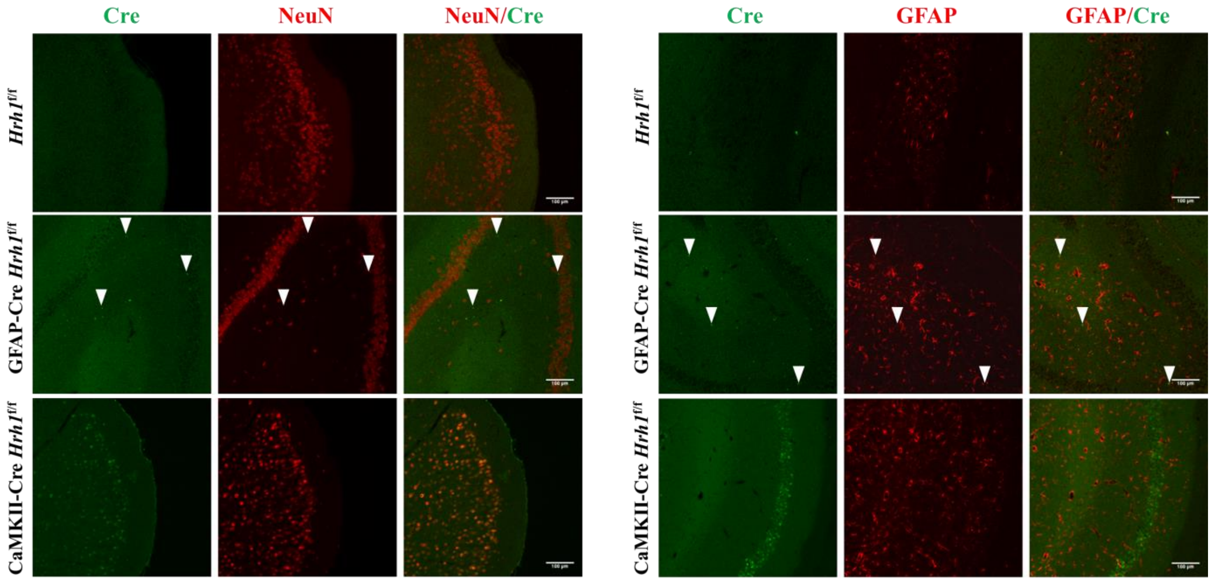

Cell-specific expression of Cre recombinase in cKO mice

Representative immunohistochemical images of Cre recombinase expression in the hippocampus and cortex of cKO and control mice. Neurons were visualised with an antibody against the neuronal marker NeuN, and astrocytes were identified with anti-GFAP (both in red). Cre-recombinase was detected with anti-Cre recombinase antibody (green). Cre-recombinase was expressed in neurons of CaMKII-Cre mice and in astrocytes of GFAP-Cre mice, respectively, but not in controls. Scale bar, 100 µm. n = 5.

Supplementary Figure S3

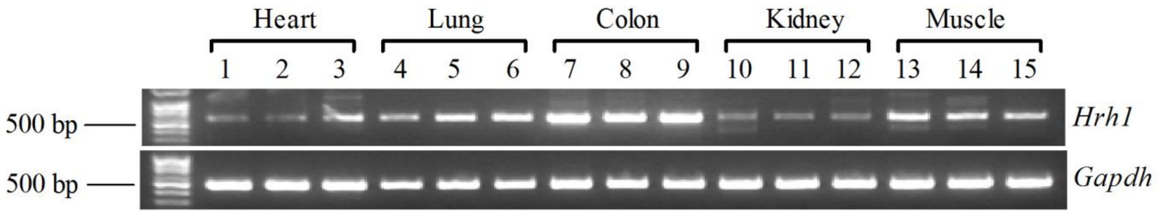

***Hrh1* expression in various tissues**

RT-PCR detection of *Hrh1* in mouse heart, lung, colon, kidney, and gastrocnemius muscle. Lanes 1, 4, 7, 10 and 13; *Hrh1* $^{f/f}$. Lanes 2, 5, 8, 11 and 14; CaMKII-Cre *Hrh1* $^{f/f}$. Lanes 3, 6, 9, 12 and 15; GFAP-Cre *Hrh1* $^{f/f}$.Glyceraldehyde-3-phosphate dehydrogenase (*Gapdh*) served as an internal control. *Hrh1*: histamine H_1 receptor.

Supplementary Table S1 Concentrations of monoamine neurotransmitters measured in selected brain regions

Brain region	Neurotransmitter	Concentration [pM/mg tissue]		
		Hrh1 f/f	GFAP-Cre *Hrh1* f/f	CaMKII-Cre *Hrh1* f/f
**Hypothalamus**	3-MT	0.02 ± 0.022	0.036 ± 0.053	0.08 ± 0.129
	5-HIAA	4.59 ± 0.959	6.73 ± 1.091	7.27 ± 2.889
	5-HT	7.76 ± 4.006	11.94 ± 3.083	8.97 ± 2.025
	DA	2.58 ± 1.052	3.57 ± 0.468	2.85 ± 0.726
	DOPAC	0.56 ± 0.245	0.69 ± 0.198	0.75 ± 0.193
	HVA	0.9 ± 0.258	1.38 ± 0.4	1.38 ± 0.434
	NE	15.73 ± 5.316	20.36 ± 3.614	18.2 ± 1.021
**Hippocampus**	3-MT	n. d.	n. d.	n. d.
	5-HIAA	5.17 ± 2.465	4.67 ± 0.874	4.79 ± 0.971
	5-HT	3.73 ± 0.648	5.07 ± 0.993	4.62 ± 1.115
	DA	0.29 ± 0.143	0.31 ± 0.058	0.24 ± 0.037
	DOPAC	0.04 ± 0.033	0.07 ± 0.028	0.1 ± 0.023
	HVA	0.31 ± 0.201	0.29 ± 0.13	0.32 ± 0.147
	NE	6.37 ± 1.606	7.08 ± 1.841	5.72 ± 0.851
**Prefrontal cortex**	3-MT	0.13 ± 0.207	0.07 ± 0.135	0.23 ± 0.357
	5-HIAA	5.31 ± 3.815	4.69 ± 1.07	5.12 ± 1.481
	5-HT	2.97 ± 0.454	4.81 ± 1.145	5.15 ± 2.319
	DA	0.8 ± 0.504	0.93 ± 0.26	1.08 ± 0.327
	DOPAC	0.27 ± 0.098	0.3 ± 0.13	0.54 ± 0.041 **
	HVA	2.23 ± 2.58	1.17 ± 0.486	1.45 ± 0.601
	NE	7.26 ± 2.467	9.81 ± 2.587	8.33 ± 3.592
**Cortex**	3-MT	n. d.	n. d.	0.02 ± 0.042
	5-HIAA	2.41 ± 1.286	1.96 ± 0.427	2.61 ± 1.317
	5-HT	2.0 ± 0.213	2.62 ± 0.617	3.4 ± 2.651
	DA	0.27 ± 0.122	0.22 ± 0.028	0.34 ± 0.16
	DOPAC	0.22 ± 0.164	0.11 ± 0.043	0.17 ± 0.077
	HVA	0.62 ± 0.401	0.51 ± 0.18	0.59 ± 0.399
	NE	5.92 ± 1.682	6.95 ± 2.122	7.23 ± 4.373
**Cerebellum**	3-MT	n. d.	n. d.	0.01 ± 0.019
	5-HIAA	1.81 ± 0.898	1.53 ± 0.504	1.68 ± 0.703
	5-HT	0.59 ± 0.238	0.92 ± 0.421	1.58 ± 1.078
	DA	0.13 ± 0.058	0.147 ± 0.011	0.2 ± 0.097
	DOPAC	0.04 ± 0.01	0.06 ± 0.016	0.09 ± 0.021 ***
	HVA	0.21 ± 0.171	0.2 ± 0.105	0.24 ± 0.113
	NE	5.44 ± 2.006	6.34 ± 2.14	5.83 ± 3.239
**Diencephalon**	3-MT	1.13 ± 0.649	1.57 ± 0.826	0.92 ± 0.501
	5-HIAA	6.95 ± 3.673	7.38 ± 2.53	10.23 ± 5.031
	5-HT	5.86 ± 1.983	8.72 ± 3.418	9.77 ± 4.996
	DA	24.85 ± 10.668	36.18 ± 12.95	29.81 ± 16.112
	DOPAC	2.91 ± 1.859	3.44 ± 1.855	3.21 ± 1.735
	HVA	3.98 ± 2.292	5.23 ± 2.331	4.38 ± 2.332
	NE	5.69 ± 2.022	7.08 ± 1.378	8.28 ± 3.622

n = 6. Statistical differences among the groups were assessed using one-way ANOVA with Tukey's post-hoc test (** $p < 0.01$ and *** $p < 0.001$); n.d. = not detected. 3-MT: 3-methoxytyramine, 5-HIAA: 5-hydroxyindoleacetic acid, 5-HT: serotonine, DA: dopamine, DOPAC: 3,4-Dihydroxyphenylacetic acid , HVA: homovanillic acid, NE: norepinephrine.
